# Reward Feedback Mechanism in Virtual Reality Serious Games in Interventions for Children With Attention Deficits: Pre- and Posttest Experimental Control Group Study

**DOI:** 10.2196/67338

**Published:** 2025-02-24

**Authors:** Hao Fang, Changqing Fang, Yan Che, Xinyuan Peng, Xiaofan Zhang, Di Lin

**Affiliations:** 1 School of Art & Design Wuhan Institute of Technology Wuhan China; 2 Engineering Research Center for Big Data Application in Private Health Medicine of Fujian Universities Putian University Putian China; 3 School of Arts and Communication China University of Geosciences Wuhan China; 4 Department of Psychiatry Tongji Hospital, Tongji Medical College Huazhong University of Science and Technology Wuhan China

**Keywords:** serious games, virtual reality, attention deficit, inhibitory control ability, reward feedback

## Abstract

**Background:**

Virtual reality (VR) serious games, due to their high level of freedom and realism, influence the rehabilitation training of inhibitory control abilities in children with attention-deficit/hyperactivity disorder (ADHD). Although reward feedback has a motivating effect on improving inhibitory control, the effectiveness and differences between various forms of rewards lack empirical research.

**Objective:**

This study aimed to investigate the effectiveness of different forms of reward feedback on the inhibitory control abilities of children with attention deficits in a VR serious game environment.

**Methods:**

This study focuses on children who meet the diagnostic criteria for ADHD tendencies, using a 2 (material rewards: coin reward and token reward) × 2 (psychological rewards: verbal encouragement and badge reward) factorial between-subject design (N=84), with a control group (n=15) for pre- and posttest experiments. The experimental group received VR feedback reinforcement training, while the control group underwent conventional VR training without feedback. The training period lasted 0.5 months, with each intervention session lasting 25 minutes, occurring twice daily with an interval of at least 5 hours for 28 sessions. Before and after training, the Swanson, Nolan, and Pelham, Version IV Scale (SNAP-IV) Scale, stop signal task, inhibition conflict task, and Simon task were administered to assess the hyperactivity index and the 3 components of inhibitory control ability. The pretest included the SNAP-IV Scale and 3 task tests to obtain baseline data; the posttest involved repeating the above tests after completing all training. Data were entered and analyzed using SPSS (IBM) software. Independent sample *t* tests were performed on the experimental and control groups’ pre- and posttest task results to determine whether significant differences existed between group means. Paired sample *t* tests were also conducted on the SNAP-IV Scale’s pre- and posttest results to assess the intervention effect’s significance.

**Results:**

Reward feedback was more effective than no reward feedback in improving behaviors related to attention deficits in children. Material rewards showed significant effects in the Stop-Signal Task (*F*_1_=13.04, *P*=.001), Inhibition Conflict Task (*F*_1_=7.34, *P*=.008), and SNAP-IV test (*F*_1_=69.23, *P*<.001); mental rewards showed significant effects in the Stop-Signal Task (*F*_1_=38.54, *P*<.001) and SNAP-IV test (*F*_1_=70.78, *P*<.001); the interaction between the 2 showed significant effects in the Stop-Signal Task (*F*_1_=4.47, *P*=.04) and SNAP-IV test (*F*_1_=23.85, *P*<.001).

**Conclusions:**

Combining material and psychological rewards within a VR platform can effectively improve attention-deficit behaviors in children with ADHD, enhancing their inhibitory control abilities. Among these, coin rewards are more effective than token rewards, and verbal encouragement outperforms badge rewards. The combined feedback of coin rewards and verbal encouragement yields the most significant improvement in inhibitory control abilities.

## Introduction

Attention-deficit/hyperactivity disorder (ADHD) is a common neurodevelopmental disorder in children, with primary clinical symptoms including inattention and behaviors related to attention deficits [1]. Current research indicates that attention deficits in patients with ADHD are primarily caused by damage or deficiencies in the inhibitory control centers. This damage leads to impairments in executive function, which is a major cause of attention-deficit behaviors in children [2]. With the development of digital health care, virtual reality (VR) technology, characterized by immersion, interactivity, and user engagement with the environment and narrative, has emerged as a promising tool for ADHD rehabilitation training in children [3-6]. Previous studies have demonstrated the feasibility of using VR in treating children with ADHD [7-12]. However, these studies remain in the experimental and exploratory phases, particularly regarding the psychological mechanisms related to inhibitory control [13,14]. Therefore, it is crucial to study the effects of different mechanisms on children’s inhibitory control abilities within a VR environment.

Studies have found that inhibitory control interventions typically use reinforcers during training. Using rewards as reinforcers can normalize the inhibitory control of children and adolescents with ADHD to a manageable baseline level. The reward mechanism serves as a supplementary intervention for ADHD, offering a potential approach to improving inhibitory control in affected children [15]. Feedback is also considered a part of reinforcement, as it is real-time and continuous, helping players continuouly improve their behavior. Rewards, on the other hand, are provided after a summary of performance and serve as an incentive to reinforce long-term participation and progress. Specifically, reward feedback involves motivating children toward desired goals through external targets outside of the task [16], such as parental recognition, badges, or praise. By using external rewards to achieve objectives, correct behaviors are repeatedly practiced and reinforced, guiding and strengthening proper awareness to achieve the goal of behavioral training. This method has proven to be highly effective in the rehabilitation training of children with ADHD [17,18]. Existing studies on the impact of reward feedback on children’s cognitive behavior choices focus largely on factors such as the form, timing, content, and conditions of the feedback [19]. The most frequently examined aspect is the form of reward, whether material rewards, social rewards, activity-based rewards, or token rewards, on cognitive behavior choices of children with ADHD. In multimedia learning environments, the introduction of various reward forms such as electronic badges, points, coins, verbal praise, and animated expressions has enriched the feedback system for children [20-24]. However, few studies have further refined the comparison of different reward feedback characteristics to determine whether there are differences in their impact on inhibitory control.

In VR training environments, the forms of reward feedback are also highly diverse. Covington and Mueller [25] have noted the use of virtual currencies, electronic badges, points, visual feedback, auditory feedback, and combined visual-auditory feedback (eg, graphics, animations, sound effects, and graphic-sound combinations) in training environments. Among these, point-based feedback is considered the most representative form of material reward feedback. Points can quantitatively reflect students’ cumulative learning behaviors and outcomes. On various platforms, points often appear in the form of coins, diamonds, stars, or small red flowers. Mental reward feedback is primarily provided through evaluations from parents, teachers, or psychological experts, with feedback presented as verbal assessments of overall status or positive reinforcement [26]. In addition, badges are seen as symbols or markers of achievement or skill, reflecting the holder’s training habits and serving as a tool for motivation and personal habit development. Therefore, further exploration of feedback optimization and innovation in VR environments, especially regarding physiological and dynamic feedback, remains of significant importance for more effective ADHD intervention training [27].

Based on the aforementioned studies, researchers have identified deficits in self-control abilities among children with ADHD, specifically in maintained response inhibition, dominant response inhibition, and interference response inhibition [28]. The high level of freedom and complexity in VR platforms may exacerbate these issues and increase the difficulty of task selection and judgment. The realism and diversity in virtual environments cause children with ADHD to overly focus on stimuli that attracts them, making it difficult to concentrate on targets, with unclear reward feedback guidance [13,15,16,23]. At the same time, current research focuses on the presence of reward feedback and the differential effects of material versus mental feedback on inhibitory control [29,30]. However, few studies have examined the differences between various forms of reward feedback in VR environments. This study aims to fill this research gap by conducting a content analysis of existing reward feedback intervention forms, designing different levels of independent variables, and mapping them into the VR environment to explore the effects of various reward feedback forms on the inhibitory control abilities of children with attention deficits.

## Methods

### Participants

The experiment initially contacted the parents of 150 children to complete the Swanson, Nolan, and Pelham, Version IV Scale (SNAP-IV) Scale (with scores ranging from 0 to 3; a score greater than 1.6 indicates a diagnosis of ADHD tendencies). Based on the scale, 112 children were selected, with 5- to 6-year-old children showing ADHD tendencies chosen as the study participants (Multimedia Appendix 1). The experiment was conducted in person, with the instructor introducing the experimental procedure and requirements to the participants. After signing the informed consent form, the instructor accompanied the participants throughout the entire experiment. Before the experiment, none of the participants had undergone any training, so there was no consideration of excluding participants with higher familiarity.

In this experiment, the independent variables were the forms of material and mental reward feedback in the VR platform. A 2 (material rewards: coins and tokens) × 2 (mental rewards: badges and verbal expressions) factorial design was used, with a control group and pre- and posttests. The experimental group (N=84) participated in VR feedback–enhanced training, while the control group (n=15) received regular VR training without feedback (correct or incorrect). Before and after the training, participants completed the SNAP-IV Scale, Stop-Signal Task, Inhibition Conflict Task, and Simon Task tests. The total training duration was 0.5 months, with each intervention session lasting 25 minutes, conducted twice per day with an interval of more than 5 hours between sessions, for a total of 28 sessions. Outcome variables included the error rate as an indicator of sustained response inhibition deficits, error rate as an indicator of dominant response inhibition deficits, and error rate effect size (ie, the difference between error rates in inconsistent and consistent trials) as an indicator of interference response inhibition deficits. In addition, the ADHD index and task completion scores were evaluated.

### Experimental Materials

**Figure 1:**
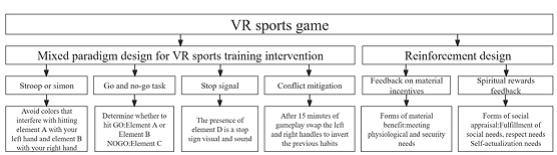
Diagram of the logic and interactive postures combining cognitive and motor training in virtual reality (VR) serious games.

The VR training game integrates cognitive and physical training, simulating a VR-based small ball game. In the game, players use a controller to hit 3 types of dynamic objects (Elements A, B, and C) launched from 4 random positions in front of them, adjusting their movements according to the launch angle [31] (Figure 1).

This study categorized VR reward feedback designs by analyzing 33 products across 9 children’s VR platforms. Results showed that material rewards in VR are primarily based on a points system, with coins accounting for 61%. While coins function similarly to other tokens, their symbolic meaning as currency requires further exploration. Thus, material rewards were divided into coin rewards and other token rewards for comparison. Verbal encouragement and badge rewards are the primary forms of psychological rewards presented by existing products. Trophies and honorary titles are similar to badges in practical terms, both serving as symbols of honor, while verbal encouragement falls under visual and auditory feedback. Therefore, this study categorizes psychological rewards into verbal encouragement and badge rewards. The study focused on the combined effects of mental and material rewards (Table 1).

**Figure 2:**
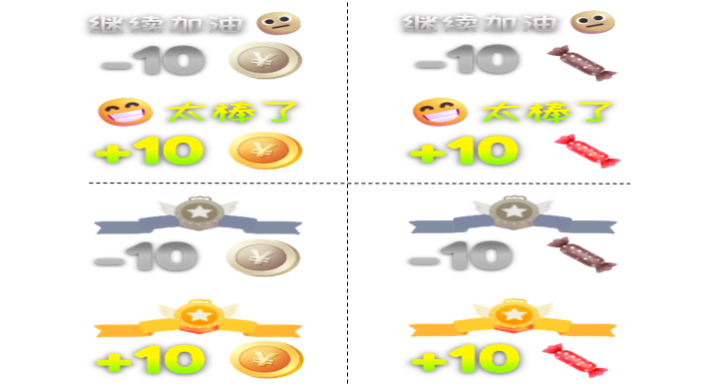
Screenshot of feedback types in serious games for the experimental group.

Based on children’s preference for bright colors, correct answers were associated with a yellow-dominant theme. The experimental group materials were designed as illustrated: from left to right and top to bottom, they represent Coin Reward and Verbal Encouragement, Token Reward and Verbal Encouragement, Coin Reward and Badge, and Token Reward and Badge (Figure 2).

**Table 1:** Classification and distribution of reward feedback on virtual reality children’s training platforms.

Reward feedback and level of incentive feedback			N^a^/(S^b^ %)
**Material rewards**			
	Coin reward	20 (61)	
	Token rewards	13 (39)	
**Mental rewards**			
	Verbal rewards	19 (58)	
	Emoji rewards	14 (42)	
	Badge rewards	17 (51)	
	Trophy rewards	3 (9)	
	Honorary title rewards	2 (6)	

^a^N: the number of rewards for feedback in 33 products across 9 VR educational resource platforms.

^b^S: the 33 products of the 9 VR educational resource platforms studied.

### Procedure and Outcome Measurement

In the pretest, the SNAP-IV Scale was first used to assess the participants’ hyperactivity index, followed by the stop signal task, Simon task, and inhibition conflict task to test their response inhibition abilities. The posttest involved repeating the same testing procedure after the completion of the VR rehabilitation training.

**Stop-Signal Task**

In this task, participants needed to complete 2 objectives. First, when symbols appeared to the left or right of a blue star, they responded by pressing the corresponding arrow key on the keyboard based on the symbol’s position relative to the star. Second, participants were instructed not to respond when a yellow sun appeared. There was a total of 120 trials, 30 of which were stop trials, accounting for 25% of the total. The error rate served as a reference indicator for maintained response inhibition deficits.

**Inhibition Conflict Task**

The task was divided into 2 parts: first, participants pressed the “B” key (blue) when a blue square appeared and pressed the “G” key (green) when a green square appeared, regardless of the spatial location of the squares. This part included 80 trials. Second, the task reversed, participants pressed the “G” key when a blue square appeared and the “B” key when a green square appeared, with 80 trials in total. The error rate in the second part served as an indicator of dominant response inhibition deficits.

**Simon Task**

Participants were instructed to ignore the spatial location of the arrows and press the key corresponding to the arrow’s direction, even if their response was incorrect, and continue to the next trial. The trials in which the spatial location and arrow direction matched were called “consistent trials,” while those in which they did not match were called “inconsistent trials.” The error rate effect size was calculated as the difference between the error rates in inconsistent and consistent trials. There were 80 inconsistent trials and 80 consistent trials, with each trial separated by a 1500-millisecond interval. The error rate effect size was used as an indicator of interference inhibition deficits.

### Statistical Analyses

To ensure the power of the experiment, we set the statistical power at 80% and the significance level at α=.05, using G*Power (Franz Faul, Edgar Erdfelder, Albert-Georg Lang, and Axel Buchnere) software to estimate the sample size, which required approximately 15 participants per group [32]. In this study, the collected data will be entered and analyzed using SPSS software. In data processing, outliers were identified using the SD method: data points deviating from the mean by more than ±2 SD were considered outliers and removed. For missing data, variables with a missing ratio exceeding 30% were deleted. Individual records with missing values were directly removed to ensure the reliability and consistency of the data analysis. To assess the effects of different reward feedback on hyperactivity indices and inhibitory control abilities in children with ADHD, a series of scientific statistical methods were applied and systematically analyzed. First, a one-way ANOVA was used to compare the pretest hyperactivity index results across different groups, verifying whether the baseline data of each group were comparable before the experiment. Subsequently, paired-sample *t* tests were used to analyze the changes in error rates before and after the intervention in both the experimental and control groups to evaluate the intervention effects of VR rehabilitation training. To further explore the effects of different reward feedback on intervention outcomes, the researchers used independent-sample *t* tests to compare the training results between the experimental groups. Simultaneously, a 2-way ANOVA was used to analyze the main effects and interactions of material and psychological rewards on the error rates of the Stop Signal Task, Inhibition Conflict Task, Simon Task, and the Snap-Iv Hyperactivity Index. The researchers further analyzed the detailed relationships between specific variables using simple effects tests for significant interactions. In addition, ANOVA was conducted to compare the mean differences between the material reward and psychological reward groups, to clarify the specific effects of different reward types.

### Ethical Considerations

The study has been reviewed and approved by the Research Ethics Committee of Wuhan University of Engineering (approval number WIT2024-001), and permission has been granted to analyze previously collected deterministic data. As the study participants are minors, their guardians signed an informed consent form after being informed about the study’s objectives, methods, and potential risks (Multimedia Appendix 2). The research data have been anonymized or deidentified and are used solely for research analysis, with strict protection of participants’ privacy and information security.

## Results

### Overview

Based on the pretest and posttest data, extreme values and invalid data were removed, and the number of participants in each group was balanced. A total of 13 participants were excluded, resulting in 99 valid data sets, with 21 participants in the Coin Reward and Badge Reward Group, 21 participants in the Token Reward and Badge Reward Group, 21 participants in the Token Reward and Verbal Encouragement Group, 21 participants in the Coin Reward and Verbal Encouragement Group, and 15 participants in the no Reward Feedback Group.

### Comparison of Material and Mental Rewards on the Hyperactivity Index of Children With ADHD

The error rates of the experimental and control groups before and after training in different tasks (Stop-Signal Task, Inhibition Conflict Task, and Simon Task) were compared. A paired-sample *t* test was conducted to compare pretest and posttest results. The results are shown in Table 2.

Before the intervention, there were no statistically significant differences between the experimental and control groups in SNAP-IV scores, Stop-Signal Task error rates, Inhibition Conflict Task error rates, and Simon Task error rates, indicating good comparability between the 2 groups. After the intervention, paired-sample *t* tests showed significant statistical differences between the pretest and posttest results of both groups (*P*<.001), indicating intervention effects. However, postintervention comparisons showed that the experimental group had significantly lower scores or error rates across all indicators compared to the control group (*P*<.001). This result suggests that interventions with reward feedback in VR training are more effective than those without reward feedback in improving ADHD-related behavioral deficits in children.

**Table 2:** Comparison of error rates in different response inhibition tasks before and after intervention between the experimental and control groups (based on paired-samples *t* test).

			Pretest, mean (SD)		Posttest, mean (SD)		*t* test (*df*)
**SNAP-IV^a^ score**							
	Experimental group (N=84)	1.36 (0.07)		1.09 (0.09)		24.94 (83)	
	Control participants (n=15)	1.35 (0.02)		1.31 (0.02)		8.35 (14)	
**Stop signal task error rate**							
	Experimental group (N=84)	0.21 (0.01)		0.10 (0.02)		77.78 (83)	
	Control participants (n=15)	0.21 (0.01)		0.14 (0.01)		29.40 (14)	
**Suppressing conflict task error rates**							
	Experimental group (N=84)	0.27 (0.01)		0.16 (0.01)		108.27 (83)	
	Control participants (n=15)	0.27 (0.001)		0.19 (0.01)		28.77 (14)	
**Simon mission error rate**							
	Experimental group (N=84)	0.21 (0.02)		0.09 (0.01)		82.03 (83)	
	Control participants (n=15)	0.20 (0.01)		0.12 (0.003)		32.60 (14)	

^a^SNAP-IV: Swanson, Nolan, and Pelham-IV rating scales.

### Comparison of Inhibitory Control Improvement in Children With ADHD Across Different Reward Feedback Levels

Independent-sample *t* tests were conducted on pre- and posttest results of sustained response inhibition training (Stop-Signal Task), dominant response inhibition deficit training (Inhibition Conflict Task), and interference response inhibition deficit training (Simon Task) for 6 groups that received different reward feedback combinations. The results are mentioned in Tables 3 and 4.

**Table 3:** Results of 2-way ANOVA on the effects of reward types on different response Inhibition Tasks and the SNAP-IV^a^ Task.

Source of variation			Square sum		*df*		Mean square		*F* test (*df*)		*P* value		*Partial Eta square*
**Stop signal task (*R*²=0.41)**													
	Intercept	0.83		1		0.83		4837.32 (1)		<.001		0.98	
	Material	0.002		1		0.002		13.04 (1)		.001		0.14	
	Mental	0.01		1		0.01		38.54 (1)		<.001		0.33	
	Material and mental	0.001		1		0.001		4.47 (1)		.04		0.05	
	Inaccuracies	0.01		80		0		N/A^b^		N/A		N/A	
**Conflict suppression task (*R*²=0.24)**													
	Intercept	2.08		1		2.08		18,821.42 (1)		<.001		1.00	
	Material	0.001		1		0.001		7.34 (1)		.008		0.08	
	Mental	0		1		0		3.23 (1)		.08		0.04	
	Material and mental	0		1		0		0.46 (1)		.50		0.01	
	Inaccuracies	0.01		80		0		N/A		N/A		N/A	
**Simon mission (*R*²=0.08)**													
	Intercept	0.63		1		0.63		3,752.46 (1)		<.001		0.98	
	Material	0		1		.00		2.42 (1)		.12		0.03	
	Mental	0		1		.00		1.17 (1)		.28		0.01	
	Material and mental	0		1		.00		1.83 (1)		.18		0.02	
	Inaccuracies	0.01		80		.00		N/A		N/A		N/A	
**SNAP-IV task (*R*²=0.672)**													
	Intercept	101.90		1		101.90		37,599.07 (1)		.00		1.00	
	Material	0.19		1		0.19		69.23 (1)		.00		0.46	
	Mental	0.19		1		0.19		70.78 (1)		.00		0.47	
	Material and mental	0.07		1		0.07		23.85 (1)		.00		0.23	
	Inaccuracies	0.22		80		0.003		N/A		N/A		N/A	

^a^SNAP-IV: Swanson, Nolan, and Pelham-IV rating scales.

^b^N/A: not available.

**Table 4:** Results of one-way ANOVA on the effects of reward types on task error rates (within the experimental group: material vs psychological rewards).

			Square sum	*df*	Mean square	*F* test	*P* value
**Stop signal task**							
	**Material**						
		Comparison	0.002	1	0.002	13.04 (1)	.001
		Inaccuracies	0.01	80	0	N/A^a^	N/A
	**Mental**						
		Comparison	0.01	1	0.01	38.54 (1)	<.001
		Inaccuracies	0.01	80	0	N/A	N/A
**Conflict suppression task**							
	**Material**						
		Comparison	0.001	1	0.001	7.34 (1)	.008
		Inaccuracies	0.01	80	0	N/A	N/A
	**Mental**						
		Comparison	0	1	0	3.23 (1)	.08
		Inaccuracies	0.01	80	0	N/A	N/A
**Simon mission**							
	**Material**						
		Comparison	0.001	1.00	0	3.51 (1)	.07
		Inaccuracies	0.02	80	0	N/A	N/A
	**Mental**						
		Comparison	0.000	1	0	2.04 (1)	.16
		Inaccuracies	0.02	80	0	N/A	N/A
**SNAP-IV^b^ task**							
	**Material**						
		Comparison	0.19	1.00	0.19	69.23 (1)	<.001
		Inaccuracies	0.22	80	0.003	N/A	N/A
	**Mental**						
		Comparison	0.19	1	0.19	70.78 (1)	<.001
		Inaccuracies	0.22	80	0.003	N/A	N/A

^a^N/A: not available.

^b^SNAP-IV: Swanson, Nolan, and Pelham-IV rating scales.

### Analysis of Stop-Signal Task Error Rate

The dependent variable was the Stop-Signal Task error rate, and the independent variables were material and mental rewards. A 2-way ANOVA was performed. As shown in Table 3, the 2-way ANOVA revealed that material rewards had a significant effect on Stop-Signal Task error rates (*F*_1_=13.04, *P*=.001), indicating a main effect of material rewards on error rates. Mental rewards also showed a significant effect (*F*_1_=38.54, *P*<.001), suggesting a main effect of mental rewards on Stop-Signal Task error rates. Furthermore, the interaction between material and mental rewards was significant (*F*_1_=4.47, *P*=.04).

A comparison of the error rates between the material reward and psychological reward groups reveals (Multimedia Appendix 3) that in both material and psychological rewards, the verbal encouragement group outperforms the badge group (0.082<0.106 and 0.099<0.111), and the coin group overall outperforms the token group (0.106<0.111 and 0.082<0.099).

From Table 4, ANOVA analysis showed that the differences within the material and mental reward groups were statistically significant. In both the coin and token groups, the mean error rates in the Stop-Signal Task differed significantly between the badge and verbal expression groups. Further simple effects tests show that the coin verbal encouragement group performs better than the coin expression group and the coin badge group (the average scores for the verbal encouragement groups in both reward conditions were 0.082 and 0.099, respectively), with the badge group showing slightly poorer improvement.

### Analysis of Inhibition Conflict Task Error Rate Effect

The dependent variable was the error rate in the Inhibition Conflict Task, and the independent variables were material and mental rewards. A 2-way ANOVA was conducted. As shown in Table 3, the analysis revealed that material rewards had a significant effect (*F*_1_=7.34, *P*<.001), indicating a main effect of material rewards on the error rate in the Inhibition Conflict Task. Mental rewards did not show a significant effect (*F*_1_=3.23, *P*=.08), indicating that the main effect of mental rewards on the error rate does not exist. In addition, the interaction between material and mental rewards was also not significant (*F*_1_=0.46, *P*=.50).

A comparison of the material and psychological rewards reveals (Multimedia Appendix 3) that in both material and psychological rewards, the verbal encouragement group outperforms the badge group (0.153<0.156 and 0.158<0.163). ANOVA results in Table 4 show that the differences within the material reward groups were statistically significant (*P*=.008), with the coin group (mean 0.15) outperforming the token group (mean 0.16). Although the differences within the mental reward groups were not statistically significant (*P*=.08), the verbal expression group had a better overall performance than the badge group. Both material and mental rewards were more effective than the control group (mean 0.18), confirming the effectiveness of reward feedback.

### Analysis of Simon Task Error Rate Effect Size

The dependent variable was the error rate effect size in the Simon Task, and the independent variables were material and mental rewards. A 2-way ANOVA was conducted. As shown in Table 3, material rewards did not show a significant effect (*F*_1_=2.42, *P*=.12), indicating that there is no main effect of material rewards on the error rate effect size in the Simon Task. Mental rewards also did not show a significant effect (*F*_1_=1.17, *P*=.28), indicating that there is no main effect of mental rewards on the error rate effect size. In addition, the interaction between material and mental rewards was not significant either (*F*_1_=1.83, *P*=.18).

A comparison of material and psychological rewards (Multimedia Appendix 2) shows that, in terms of means, the coin verbal encouragement group (mean 0.08) < coin badge group (mean 0.08) < token verbal encouragement group (mean 0.09) < token badge group (mean 0.10), with the coin group outperforming the token group overall, and the verbal encouragement group outperforming the badge group. The coin group overall outperformed the token group, and the verbal expression group outperformed the badge group. However, ANOVA results showed that the differences within the material and mental reward groups were not statistically significant (as shown in Table 4).

### Analysis of SNAP-IV Hyperactivity Index

The dependent variable was the SNAP-IV hyperactivity index, and the independent variables were material and mental rewards. A 2-way ANOVA was conducted to study the relationship between material and mental rewards and the hyperactivity index. As shown in the table, material rewards had a significant effect (*F*_1_=69.23, *P*<.001), indicating that material rewards have a main effect on the hyperactivity index. Mental rewards also showed a significant effect (*F*_1_=70.78, *P*<.001), indicating that mental rewards have a main effect on the hyperactivity index. In addition, the interaction between material and mental rewards was significant (*F*_1_=23.85, *P*<.001).

A comparison between material and mental rewards shows that the verbal expression group outperformed the badge group for both reward types, and the coin group outperformed the token group overall. ANOVA analysis revealed statistically significant differences within the material and mental reward groups, indicating that the mean hyperactivity index in the badge and verbal expression groups differed between the coin and token groups. Further simple effects tests showed that in both material and psychological rewards, the verbal encouragement group significantly outperformed the badge group, with differences being statistically significant (*P*<.001).

## Discussion

### Principal Findings

This study found that VR intervention training with reward feedback significantly improved inhibitory control in children with ADHD, with material rewards proving more effective than psychological rewards. Specifically, coin rewards were more effective than token rewards, and verbal encouragement outperformed badge rewards. The combination of material and psychological rewards had the most optimal effect on enhancing inhibitory control, particularly the combination of coin rewards and verbal encouragement, which significantly improved the 3 main components of sustained response inhibition, dominant response inhibition, and interference response inhibition. The experimental results are consistent with the conclusions of Sagvoldon’s dynamic developmental theory model of ADHD (2005), which posits that ADHD involves a complex interaction between genetic predisposition and environmental influences [33]. Therefore, given the conflict between the flexibility of VR environments and the developmental characteristics of children with ADHD, incorporating the motivating effects of reward feedback into intervention training is both important and necessary.

### Comparison With Previous Work

The results indicate that material rewards had significant main effects in the Stop-Signal Task, Inhibition Conflict Task, and SNAP-IV tests, with coin rewards proving significantly more effective than token rewards. The experimental study found that material rewards increase individuals’ focus and effort on tasks, thereby improving inhibitory control. When individuals are aware that they will receive a tangible material reward after completing a task, they are more motivated to suppress impulses and temptations, focusing on achieving the goal. This may be because material rewards are directly related to individuals’ physiological needs and desires, which activate the reward system, thus enhancing cognitive control. This finding is consistent with Fosco et al [34], who demonstrated that coin rewards significantly improve task performance in children with ADHD. In addition, studies using functional magnetic resonance imaging (fMRI) have found that material rewards enhance activity in the prefrontal cortex, particularly in the control networks related to inhibitory control. This suggests that material rewards actively improve individuals’ inhibitory control by activating neurobiological mechanisms. *Meyer* [35] further demonstrated that increasing coin rewards more effectively enhances task performance in children with ADHD, particularly by improving attention and inhibitory abilities. However, the difference in effectiveness between coin and token rewards may be due to several factors. Studies have suggested that while both coin and token rewards operate effectively in symbolic economic environments, the actual use of money is less likely. However, the stronger monetary significance of coins makes them more effective in encouraging correct behavior in children. This type of reinforcement has been shown to be effective in children with similar disorders, such as autism spectrum disorder. This finding is consistent with the current study, where the controlling significance of coin rewards prompted children to adjust their learning behavior to obtain rewards [36].

In addition, the study found that mental rewards did not show significant main effects in the Inhibition Conflict Task and Simon Task. Children with ADHD may exhibit differences in their sensitivity to rewards and motivation levels [37], being more responsive to immediate and tangible material rewards, while their response to abstract mental rewards may be weaker. Material rewards typically have clear and immediate effects, such as snacks or toys. Mental rewards, such as praise or recognition, tend to be more indirect and abstract, and may not be as immediately noticeable as material rewards. For children with ADHD, immediacy and visibility are crucial to the effectiveness of rewards, making mental rewards relatively less effective. This may also be related to the characteristics of children with ADHD, such as difficulty with attention, impulsivity, and delayed gratification [38-41]. ADHD is associated with abnormalities in the dopamine system, which plays a key role in reward processing and motivation regulation. Mental rewards typically involve internal satisfaction and motivational activation, but children with ADHD may have dopamine regulation deficits, leading to a diminished response to mental rewards.

The study further examined the interaction between material and mental rewards on inhibitory control abilities. The interaction effect was significant only in the Stop-Signal Task and SNAP-IV test, with the verbal expression group outperforming the badge group. The combination of verbal expression and coin rewards was superior to the combination of badges and tokens, indicating that combining material and mental rewards can enhance the motivational effect for children with ADHD. Material rewards provide direct external motivation, while mental rewards offer intrinsic and social motivation. The 2 complement each other, enhancing the responses of children with ADHD to rewards. Research has shown that compared with token-based material rewards, the monetary nature of coin rewards can stimulate higher training motivation. This aligns with previous studies using fMRI and event-related potentials (ERPs), which demonstrated that monetary rewards more significantly activate brain regions involved in reward feedback processes [42,43]. In the combination of token rewards and verbal encouragement, verbal encouragement dominated the reward effect, contributing to a gradual and lasting improvement in intervention outcomes [44]. The superiority of verbal encouragement over badges is consistent with BERNIS’s findings, where smiley face (expression) feedback showed a significant negative correlation with P2, P3, and feedback-related negativity amplitudes, indicating that smiley faces as reinforcers were not particularly effective. In addition, related studies have shown that children with ADHD lack awareness of others’ emotions [45,46], similar to children with autism spectrum disorder. Patients with ADHD often have difficulty understanding social cues [47]. While patients with ADHD may have social interest, they often struggle to evaluate social feedback, such as facial expressions [36]. In contrast, verbal encouragement provides more positive informational value, enhancing children’s sense of competence and, in turn, increasing their intrinsic motivation [42], which positively impacts task performance. This further explains why verbal encouragement outperforms badges.

### Strengths and Limitations

The results of this study not only validate the core principles of the Health Behavior Change Theory and Gamification Theory but also provide important empirical support for their development. The Health Behavior Change Theory emphasizes the key role of external incentives and internal motivation in shaping behavior [48,49]. This study, through the design of material rewards (such as coins) and psychological rewards (such as verbal encouragement) in VR training, provided immediate external incentives, enhanced children’s sense of competence and intrinsic motivation, and significantly improved their inhibitory control, thereby validating the positive impact of immediate feedback on behavioral self-regulation. Gamification Theory advocates motivating users to engage and change behavior through game elements such as reward mechanisms and immediate feedback. This study shows that the combination of coins and verbal encouragement had the most effective motivational impact, not only increasing task interest but also enhancing children’s sense of achievement, reflecting the core principles of competence, relatedness, and autonomy in Gamification Theory.

In addition, this study is the first to systematically compare different reward forms (such as coins vs tokens and verbal encouragement vs badges) and their combined effects. By integrating existing fMRI and ERP studies, it reveals the neurobiological basis of reward mechanisms in enhancing inhibitory control through the activation of the prefrontal cortex. This further refines the theoretical framework and fills the gap in research on the effects of reward mechanisms in ADHD interventions. The findings suggest that for special populations (such as children with ADHD), it is necessary to adjust the design of social incentives, prioritizing more direct forms of motivation such as verbal encouragement. At the same time, the role of immediate rewards in promoting long-term behavior change should be emphasized, providing theoretical support and design insights for health behavior interventions in these populations [50,51].

By integrating VR technology to provide an immersive intervention environment, multidimensionally assessing children’s inhibitory control (such as sustained response inhibition, dominant response inhibition, and interference response inhibition), and exploring the neurobiological mechanisms of reward feedback in cognitive-behavioral improvement, this study offers new perspectives from both practical and theoretical levels. It not only advances the development of Health Behavior Change Theory and Gamification Theory but also provides important references for the design of educational games and VR intervention tools for children.

However, the study should also address several influencing factors. First, the characteristics and environmental backgrounds of children with ADHD may vary, resulting in individual differences in their responses to rewards. We attempted to recruit a diverse sample to address this, but individual variability could still influence the results. Future studies should control for these factors or explore how they mediate reward effectiveness. Second, the reward culture within the family, school, and social environments could affect how children respond to reward feedback. This study did not account for these external influences, which may limit the generalizability of the findings. In future research, it would be helpful to assess these cultural influences and their potential impact on reward responses [52]. Third, the study sample was relatively small and homogeneous regarding age and gender. A more extensive and diverse sample, including a wider age range and varying levels of ADHD severity, could provide a more comprehensive understanding of how rewards affect inhibitory control. In addition, future studies should explore the effects of different reward intensities and their long-term impact on behavior. Fourth, this study did not use precise neural monitoring instruments, such as electroencephalogram or eye-tracking devices, to monitor children’s attention control during the intervention. Future studies should incorporate these tools to provide a more accurate assessment of the neurobiological mechanisms underlying the effects of reward feedback.

### Conclusion

This empirical study explored the design of reward feedback to enhance inhibitory control in children with attention deficits. It examined the effects of VR interventions with and without reward feedback in ADHD training, as well as the influence of material and mental rewards on 3 main components of ADHD in children (sustained response inhibition deficit, dominant response inhibition deficit, and interference response inhibition deficit). The study further investigated the impact of the levels of independent variables on inhibitory control ability.

The study yielded the following conclusions: (1) a VR intervention platform using both material and mental rewards as feedback can effectively improve sustained response inhibition deficits, dominant response inhibition deficits, and interference response inhibition deficits in children with ADHD, thereby enhancing overall inhibitory control abilities; (2) material reward feedback using coin rewards is more effective in improving inhibitory control abilities in children than token (candy) rewards; (3) mental reward feedback using verbal encouragement is more effective in improving inhibitory control abilities than badge rewards; and (4) the combination of coin rewards and verbal encouragement feedback yields the best results in enhancing children’s inhibitory control abilities.

## Appendix

Multimedia Appendix 1 SNAP-IV (Swanson, Nolan, and Pelham, Version IV Scale) scale (parent version; 2018).

Multimedia Appendix 2 Informed Consent Form.

Multimedia Appendix 3 Comparison of error rates between material and spiritual rewards.

## Abbreviations

ADHD: attention-deficit/hyperactivity disorder
ERP: event-related potential
fMRI: functional magnetic resonance imaging
SNAP-IV: Swanson, Nolan, and Pelham, Version IV Scale
VR: virtual reality

## Data Availability

All data generated or analyzed during this study are included in this published article (and Multimedia Appendices 1-3 information files).
