# Supplementary material for: Reward Feedback Mechanism in Virtual Reality Serious Games in Interventions for Children With Attention Deficits: Pre- and Posttest Experimental Control Group Study
Source: JMIR Serious Games. 2025 Feb 24;13:e67338. doi: 10.2196/67338 (PMC11894355; doi:10.2196/67338)
Supplement: Multimedia Appendix 1 [file games_v13i1e67338_app1.docx]

Appendix1. SNAP-IV Scale (Parent Version) [2018]

*1. Frequently loses temper

·Never or rarely occurs

·Occasionally occurs, with little or no impact

·Common, with moderate impact on self or others

·Very common, occurring once or more daily, with severe impact on self or others

·Never or rarely occurs

*2. Frequently argues with adults

·Never or rarely occurs

·Occasionally occurs, with no or minimal impact

·Common, with moderate impact on oneself or others

·Very common, occurring once or more per day, causing significant impact on oneself or others

*3. Frequently defies or refuses to comply with adult demands or rules

·Never or rarely occurs

·Occasionally occurs, with no or minimal impact

·Common, with moderate impact on oneself or others

·Very common, occurring once or more per day, causing significant impact on oneself or others

*4. Frequently engages in actions that intentionally provoke others

·Never or rarely occurs

·Occasionally occurs, with no or minimal impact

·Common, with moderate impact on oneself or others

·Very common, occurring once or more per day, causing significant impact on oneself or others

*5. Frequently blames others for one’s own mistakes or failures

·Never or rarely occurs

·Occasionally occurs, with no or minimal impact

·Common, with moderate impact on oneself or others

·Very common, occurring once or more per day, causing significant impact on oneself or others

*6. Frequently overly sensitive or easily irritated by others

·Never or rarely occurs

·Occasionally occurs, with no or minimal impact

·Common, with moderate impact on oneself or others

·Very common, occurring once or more per day, causing significant impact on oneself or others

*7. Frequently angry or enraged

·Never or rarely occurs

·Occasionally occurs, with no or minimal impact

·Common, with moderate impact on oneself or others

·Very common, occurring once or more per day, causing significant impact on oneself or others

*8. Frequently holds grudges or seeks revenge

·Never or rarely occurs

·Occasionally occurs, with no or minimal impact

·Common, with moderate impact on oneself or others

·Very common, occurring once or more per day, causing significant impact on oneself or others

*9. Often fails to pay attention to detail or makes careless errors while completing tasks

·Never or rarely occurs

·Occasionally occurs, with no or minimal impact

·Common, with moderate impact on oneself or others

·Very common, occurring once or more per day, causing significant impact on oneself or others

*10. Difficulty maintaining attention during tasks or play

·Never or rarely occurs

·Occasionally occurs, with no or minimal impact

·Common, with moderate impact on oneself or others

·Very common, occurring once or more per day, causing significant impact on oneself or others

*11. Appears not to be listening when spoken to

·Never or rarely occurs

·Occasionally occurs, with no or minimal impact

·Common, with moderate impact on oneself or others

·Very common, occurring once or more per day, causing significant impact on oneself or others

*12. Frequently fails to follow instructions consistently and is unable to complete schoolwork, chores, or tasks

·Never or rarely occurs

·Occasionally occurs, with no or minimal impact

·Common, with moderate impact on oneself or others

·Very common, occurring once or more per day, causing significant impact on oneself or others

*13. Difficulty organizing and planning tasks and activities

·Never or rarely occurs

·Occasionally occurs, with no or minimal impact

·Common, with moderate impact on oneself or others

·Very common, occurring once or more per day, causing significant impact on oneself or others

*14. Avoids tasks that require sustained mental effort (e.g., school assignments, homework)

·Never or rarely occurs

·Occasionally occurs, with no or minimal impact

·Common, with moderate impact on oneself or others

·Very common, occurring once or more per day, causing significant impact on oneself or others

*15. Frequently loses necessary items for tasks or activities (e.g., toys, schoolwork, pencils, or books)

·Never or rarely occurs

·Occasionally occurs, with no or minimal impact

·Common, with moderate impact on oneself or others

·Very common, occurring once or more per day, causing significant impact on oneself or others

*16. Easily distracted

·Never or rarely occurs

·Occasionally occurs, with no or minimal impact

·Common, with moderate impact on oneself or others

·Very common, occurring once or more per day, causing significant impact on oneself or others

*17. Frequently forgets things in daily activities

·Never or rarely occurs

·Occasionally occurs, with no or minimal impact

·Common, with moderate impact on oneself or others

·Very common, occurring once or more per day, causing significant impact on oneself or others

*18. Fidgets or squirms in seat

·Never or rarely occurs

·Occasionally occurs, with no or minimal impact

·Common, with moderate impact on oneself or others

·Very common, occurring once or more per day, causing significant impact on oneself or others

*19. Leaves seat in classroom or other situations requiring sitting

·Never or rarely occurs

·Occasionally occurs, with no or minimal impact

·Common, with moderate impact on oneself or others

·Very common, occurring once or more per day, causing significant impact on oneself or others

*20. Runs or climbs excessively in inappropriate situations

·Never or rarely occurs

·Occasionally occurs, with no or minimal impact

·Common, with moderate impact on oneself or others

·Very common, occurring once or more per day, causing significant impact on oneself or others

*21. Difficulty playing quietly or engaging in leisure activities

·Never or rarely occurs

·Occasionally occurs, with no or minimal impact

·Common, with moderate impact on oneself or others

·Very common, occurring once or more per day, causing significant impact on oneself or others

*22. Always busy, running around, with actions resembling those driven by an engine

·Never or rarely occurs

·Occasionally occurs, with no or minimal impact

·Common, with moderate impact on oneself or others

·Very common, occurring once or more per day, causing significant impact on oneself or others

*23. Talks excessively

·Never or rarely occurs

·Occasionally occurs, with no or minimal impact

·Common, with moderate impact on oneself or others

·Very common, occurring once or more per day, causing significant impact on oneself or others

*24. Interrupts others by answering questions before they are fully asked

·Never or rarely occurs

·Occasionally occurs, with no or minimal impact

·Common, with moderate impact on oneself or others

·Very common, occurring

*25. Difficulty waiting for one's turn

·Never or rarely occurs

·Occasionally occurs, with no or minimal impact

·Common, with moderate impact on oneself or others

·Very common, occurring once or more per day, causing significant impact on oneself or others

*26. Interrupts or disrupts others' conversations or activities

·Never or rarely occurs

·Occasionally occurs, with no or minimal impact

·Common, with moderate impact on oneself or others

·Very common, occurring once or more per day, causing significant impact on oneself or others
