# Supplementary material for: Reward Feedback Mechanism in Virtual Reality Serious Games in Interventions for Children With Attention Deficits: Pre- and Posttest Experimental Control Group Study
Source: JMIR Serious Games. 2025 Feb 24;13:e67338. doi: 10.2196/67338 (PMC11894355; doi:10.2196/67338)
Supplement: Multimedia Appendix 2 [file games_v13i1e67338_app2.docx]

Appendix 2. **Informed Consent Form**

Dear Subject.

We would like to invite you to participate in a study of rewarding feedback mechanisms in VR serious games for children with Attention Deficit Disorder (ADD). This informed consent form provides you with information to help you decide whether or not to participate in this study. Please take some time to read the following carefully and if you have any questions, please address them to the researcher in charge of the study.

Your participation in this study is completely voluntary. This study has been reviewed and approved by the Research Ethics Committee of Wuhan Engineering University.

Background of the study:

Virtual reality, as a new treatment modality, is becoming a trend in the rehabilitation training of ADHD children. However, ADHD children have poor self-control, and the high degree of freedom and realism of virtual reality platforms may conflict with the development of their inhibitory control. It has been demonstrated that reward feedback can motivate inhibitory ability, but the existing reward feedback design mostly draws on game mechanisms or is in the primary stage of digitisation, and there is a lack of empirical research on virtual reality, and the effectiveness and differences of reward forms still need to be analysed in depth.

Purpose of the study:

The purpose of this study is to investigate the effects of different manifestations of reward feedback on the inhibitory control ability of attention-deficit children in a virtual reality serious gaming environment, and to further analyse the effectiveness of different combinations of reward feedback forms in improving their effects.

Research Process:

This study adopted a two-factor between-subjects design of 2 (material rewards: gold rewards, token rewards) × 2 (mental rewards: verbal expression encouragement, badge rewards), and set up a control group for a pre and post-test experiment. The experimental group received intensive training with virtual reality feedback, and the control group underwent regular virtual reality training without feedback. The training period was 0.5 months, and the training duration of each intervention was 25 minutes, twice a day with an interval of more than 5 hours, for a total of 28 sessions. The SNAP-IV scale, stop-signal task, conflict inhibition task and Simon task were tested before and after the training.

Risks and discomforts of participating in the study:

There are no known risks to the psychological and physiological aspects of the research process for the participants.

Benefits of Participating in the Study:

You and your child will have the opportunity to learn about relevant attention intervention methods through virtual reality training and may benefit from the training process.

Costs associated with participation in the study:

You will not be charged any fees for this study. To compensate for the expenses of participating in the study (e.g., your travelling expenses, etc.) you will receive a gift that we have prepared for you.

Right to refuse to participate or withdraw from the study:

You may discontinue your participation at any time if you feel uncomfortable in any way during the course of your participation.

Privacy and Confidentiality Issues: Your personal information such as name, gender, etc. will be replaced with codes or numbers and will be kept strictly confidential, and any report on the results of this study will not disclose any of your personal information.

**Informed Consent Signature Page**

If you fully understand the contents of this research project and agree to participate in this study, you will sign this informed consent form in duplicate, with one copy retained by the investigator and one by the subject.

Consent Statement:

1. I acknowledge that I have read and understand the informed consent form for this research study.

2. I have had the opportunity to ask questions and all questions have been answered. 3.

3. I understand that participation in this study is voluntary. 4.

4. I can choose not to participate in this study or withdraw at any time by notifying the researcher without discrimination or retaliation, and that none of my rights will be affected as a result.

5. I have been informed that the investigators of this study and the Research Ethics Committee of Wuhan University of Engineering have the right to review the study records and related information, and I understand that any information about me will be treated confidentially.

Subject's name: _________________

Subject's signature: _________________

Subject's phone number: _________________

Date: _________ Year ____ Month ____ Day

I have accurately communicated this document to the subject, he/she has accurately read this informed consent form, and I certify that the subject was given the opportunity to ask questions. I certify that his/her consent is voluntary.

Investigator's name: _________________

Investigator's signature: _________________

Investigator's phone number: _________________

Date: _________ Year ____ Month ____ Day

Note: Witness signature is required if the subject is illiterate, or proxy signature if the subject is incapacitated.
