# Supplementary material for: Reward Feedback Mechanism in Virtual Reality Serious Games in Interventions for Children With Attention Deficits: Pre- and Posttest Experimental Control Group Study
Source: JMIR Serious Games. 2025 Feb 24;13:e67338. doi: 10.2196/67338 (PMC11894355; doi:10.2196/67338)
Supplement: Multimedia Appendix 3 [file games_v13i1e67338_app3.docx]

Appendix 3. Comparison of error rates between material and spiritual rewards

| Material | Mental | Mean | SD |
| --- | --- | --- | --- |
| Stop signal task |  |  |  |
| Coin reward | Badge rewards | 0.106 | 0.017 |
|  | Emoji rewards | 0.082 | 0.008 |
|  | Sum | 0.094 | 0.018 |
| Token rewards | Badge rewards | 0.111 | 0.013 |
|  | Emoji rewards | 0.099 | 0.012 |
|  | Sum | 0.105 | 0.014 |
| Conflict suppression task |  |  |  |
| Coin reward | Badge rewards | 0.156 | 0.010 |
|  | Emoji rewards | 0.153 | 0.009 |
|  | Sum | 0.154 | 0.010 |
| Token rewards | Badge rewards | 0.163 | 0.011 |
|  | Emoji rewards | 0.158 | 0.011 |
|  | Sum | 0.160 | 0.011 |
| Simon mission |  |  |  |
| Coin reward | Badge rewards | 0.081 | 0.007 |
|  | Emoji rewards | 0.080 | 0.011 |
|  | Sum | 0.081 | 0.009 |
| Token rewards | Badge rewards | 0.095 | 0.011 |
|  | Emoji rewards | 0.086 | 0.009 |
|  | Sum | 0.090 | 0.011 |
| SNAP-IV task |  |  |  |
| Coin reward | Badge rewards | 1.130 | 0.045 |
|  | Emoji rewards | 0.979 | 0.065 |
|  | Sum | 1.054 | 0.094 |
| Token rewards | Badge rewards | 1.169 | 0.044 |
|  | Emoji rewards | 1.129 | 0.051 |
|  | Sum | 1.149 | 0.051 |
